# Fixed versus Unfixed Combination of Topical Latanoprost/Timolol for Glaucoma: An Observational Study Investigating the Level of Adherence and Ocular Surface Health

**DOI:** 10.3390/jcm12093137

**Published:** 2023-04-26

**Authors:** Victoria Toumanidou, Asterios Diafas, Nikolaos Georgiadis, Ioannis Tsinopoulos

**Affiliations:** 1 2nd Department of Ophthalmology, Papageorgiou Hospital, Aristotle University of Thessaloniki, 56403 Thessaloniki, Greece; 2 1st Department of Ophthalmology, Ahepa Hospital, Aristotle University of Thessaloniki, 54636 Thessaloniki, Greece

**Keywords:** ocular hypertension (OHT), primary open-angle glaucoma (POAG), exfoliation glaucoma (XFG), ocular surface disease (OSD), compliance

## Abstract

Purpose: To investigate the effect of fixed combination versus concomitant unfixed topical glaucoma treatment on patients’ adherence and ocular surface health. Patients and Methods: This is a 6-month, prospective, parallel-group, observational study in patients with ocular hypertension (OHT), primary open-angle glaucoma (POAG), or exfoliation glaucoma (XFG). A total of 142 patients with similar baseline characteristics were enrolled in this study. Seventy-one patients received a Latanoprost 0.005%/Timolol 0.5% fixed combination in the evening, whereas seventy-one patients received the unfixed treatment with Latanoprost 0.005% in the evening and Timolol 0.5% twice daily. The primary outcome was the adherence rate at baseline, and at the 3- and 6-month follow-up visits. The secondary outcomes included the signs of ocular surface disease (OSD) and intraocular pressure (IOP). Results: The adherence of patients treated with the fixed combination was higher than the unfixed treatment at the 3-month (78.0% vs. 63.0%, *p* < 0.001) and at the 6-month visits (73.0% vs. 58.5%, *p* < 0.01). The Break-up Time, Schirmer test, and Van Bijsterveld score were worse in the unfixed group at baseline and all subsequent examinations (*p* < 0.05 for all comparisons). There were no differences in the mean IOP between groups at baseline, 1-, and 3-month visits. IOP appears higher in the unfixed group at 6 months (16.7 vs. 15.0 mmHg, *p* < 0.01). Conclusion: The patients with ocular hypertension and primary open-angle glaucoma treated with a fixed combination are significantly more adherent and show a healthier ocular surface than those treated with an unfixed combination. The study provides significant evidence of the benefits of fixed combination treatment.

## 1. Introduction

Glaucoma is one of the leading causes of visual impairment and blindness worldwide. Pharmacologic agents remain the gold standard for treating ocular hypertension and glaucoma, despite the significant advances in laser and surgical treatment. The most common therapeutic options are prostaglandin analogues, beta-blockers (beta-adrenergic receptor antagonists), carbonic anhydrase inhibitors (CAIs), and alpha agonists (alpha-adrenergic receptor agonists) [1].

Monotherapy remains the first line of treatment in glaucoma, although many patients require a combination of anti-glaucoma agents to achieve adequate IOP reduction. Approximately half of the patients with open-angle glaucoma need an additional anti-glaucoma agent within 2 years of initiating the glaucoma treatment [2,3].

Fixed combinations are commonly used to control intraocular pressure (IOP) instead of the single administration of the individual drug components. Several fixed-dose combinations of anti-glaucoma agents have been introduced to reduce the incidence of side effects, maximise the long-term treatment tolerance and adherence to medication, and to improve patients’ quality of life [4,5,6,7,8,9,10].

Poor adherence is one of the main reasons for therapeutic failure in glaucoma treatment. The compliance cannot be accurately evaluated, given that the percentage of patients who overestimate treatment adherence ranges between 23% and 59% [11]. An accurate and objective method to assess the treatment adherence is the electronic recording providing measurements independent of the clinical parameters. The adherence rate was measured between 76% and 86% in studies using electronic recording [12,13,14], with long-term adherence appearing significantly lower. Only 57% of patients receive more than 80% of the prescribed treatment at a 6-month follow-up visit [15].

The initiation of anti-glaucoma medication can cause many adverse events from the ocular surface, negatively impacting patient adherence [11,15,16]. Ocular surface disease (OSD) is a multifactorial disorder of the corneal and conjunctival epithelium, the lacrimal and meibomian glands. It is mainly associated with visual disorders and symptoms of ocular discomfort [17].

About 15% of the elderly population experiences some OSD symptoms [18]. Patients with ocular hypertension and glaucoma have been found to suffer from these symptoms more often. It is estimated that approximately 60% of glaucoma patients under topical medication report OSD symptoms. In addition, glaucoma also coexists in patients suffering from severe OSD, about 66% [19,20].

Fixed drug combinations have some advantages compared to combination therapy regarding the easiness of use by the patient, the prevention of drug washout, and the reduction in long-term exposure to preservatives, resulting in fewer ocular surface symptoms [21].

Although many studies have investigated the role of various anti-glaucoma drug combinations compared to the individual components, this study aims to evaluate the compliance to the Latanoprost/Timolol combination versus unfixed combination in real-world conditions, using a reliable electronic monitoring system with the patient being unaware that their compliance is being recorded. The present study also aims to assess the ocular surface health and IOP-lowering effect between these two groups.

## 2. Materials and Methods

This is a 6-month, prospective, parallel-group, observational study conducted at the Glaucoma Department of the Ophthalmology Clinic of the “AHEPA” University Hospital of Thessaloniki. The study followed the tenets of the Declaration of Helsinki and was approved by the Biomedical Ethics Committee of the Aristotle University of Thessaloniki. Written informed consent was obtained from all participants following a thorough explanation of the protocol before their enrolment in the study.

To be included in the study, the patients had to be between 21 and 80 years old with a best corrected visual acuity (BCVA) higher than 6/60. All participants were diagnosed with well-controlled ocular hypertension (OHT), primary open-angle glaucoma (POAG), or exfoliation glaucoma (XFG), and started using fixed or unfixed Latanoprost and Timolol treatment for a period between 3 and 6 months before being enrolled in the study. The average of at least two consecutive IOP measurements before treatment should have been calculated between 19 mmHg and 32 mmHg. The patients should appear to have open normal angles with a minimum IOP decrease of 20% after treatment initiation to be included in the study. The exclusion criteria were any ocular surface disease, active ocular inflammation, history of ocular trauma, steroid use in the last two months, and any contraindications for glaucoma eye drops to use. Pregnant and breastfeeding women, patients with poor treatment adherence or follow-up, contact-lens wearers, and any corneal abnormality that may affect the IOP measurement were also excluded from the study.

The participants were divided into two groups. Group A included patients treated with the fixed combination of Latanoprost/Timolol (LTFC) (Pfizer & Upjohn Co., Puurs, Belgium) once a day (8:00 p.m.). Group B patients were treated with (concomitant medications) Timolol (MSD/Vianex, Athens, Greece) twice a day (8:00 a.m., 8:00 p.m.), and Latanoprost (Pfizer & Upjohn Co., Puurs, Belgium) once a day (8:15 p.m.). Treatments in both groups included benzalkonium chloride (BAK) as preservatives.

All the demographic information and a thorough ophthalmic and systematic medical history were recorded at the baseline visit. One eye from each patient was randomly selected for the study. All patients underwent a comprehensive examination by an experienced ophthalmologist, including best-corrected visual acuity (BCVA), slit-lamp examination, central corneal pachymetry, IOP measurement, dilated fundoscopic examination, documentation of vertical cup/disc ratio, and a Humphrey visual field test. The tear film break-up time (TBUT) was calculated using the mean value of 3 consecutive measurements, whereas the Schirmer test I was also measured. The cornea and conjunctiva integrity was evaluated using fluorescein (Bausch & Lomb Ltd., London, UK) and lissamine green (Macsen Laboratories, Rajasthan, India), according to the Van Bijsterveld rating system.

The morning IOP and adverse events were recorded at 1-, 3-, and 6-month follow-up visits. The clinical signs of ocular surface disease (TBUT, Schirmer test I, and Van Bijsterveld score) and online compliance recording were also assessed at the three- and six-month visits. In addition, all patients were asked to record the time of treatment application in a personal calendar. The treatment compliance was recorded electronically with the MEMS Cap monitoring system (Medication Event Monitoring System, AARDEX Ltd., Geneva, Switzerland) without the patients being aware of being monitored for adherence. Non-adherence was defined as a failure of instillation at least once daily during the study.

In this study, the primary outcome investigated was the adherence rate in patients who received either unfixed or fixed combination treatment of Latanoprost/Timolol at 3 and 6 months after their enrolment in the study. The secondary outcomes were the OSD signs and the assessment of IOP at the time points. The adverse events, the number of patients lost to follow-up, and the frequency of missing follow-up examinations were also recorded.

The statistical analysis of our study was conducted using the Statistical Package for Social Sciences software (SPSS, version 25.0; IBM SPSS, Inc., Chicago, IL, USA). The Shapiro–Wilk test was used to test the normality of distribution for the continuous variables. The median and interquartile range (IQR) were used to present the descriptive statistics of non-normally distributed variables, whereas the mean and standard deviation (SD) were used for the normally distributed variables. The Mann–Whitney U test was used to compare the non-parametric variables, whereas the independent *t*-test was for the parametric ones. A *p*-value of less than 0.05 was considered significant.

## 3. Results

In total, 142 patients (71 patients in each group) were enrolled in the present study. The participants appear to have similar baseline characteristics regarding age, gender, pre-study treatment duration, initial diagnosis, BCVA, and corneal thickness. The study included 51 males (35.9%) and 91 females (64.1%). The median age (IQR) was 66.0 (19.0) years (range 20–88) (Table 1).

The adherence rate was significantly higher in the fixed combination group at 3 (78.0% vs. 63.0%, *p* < 0.001) and 6 months of follow-up visits (73.0% vs. 58.5%, *p* < 0.01) compared to the unfixed group.

The signs of ocular surface health were significantly better in the LTFC group at baseline and all follow-up visits. The BUT (SD) was 6.0 (4.0) s and 5.0 (2.0) s at baseline for the LTFC group and concomitant medication group, respectively (*p* < 0.01). The respective BUT was 5.0 s and 4.0 s at the 3- and 6-month visits (*p* < 0.001). The Van Bijsterveld score in the fixed combination group was 1.0 and 2.0, whereas the unfixed group was 2.0 and 3.0 at the baseline and 6-month visits, respectively. The difference between the two groups regarding the Schirmer test was similar and remained stable throughout the study. The fixed combination treatment showed 11.0 mm compared to 8.0 mm in the unfixed group at baseline (*p* < 0.001). The difference between groups was 3.0 mm at the 3-month visit and 3.5 mm at the 6-month visit (*p* < 0.01 for both time points).

The mean IOP was 15.79 mmHg in the fixed group and 15.97 mmHg in the unfixed group at baseline without significant changes at the 1- and 3-month follow-up visits. However, at the 6-month visits, the LTFC group showed a mean IOP reduction of 0.79 mmHg, whereas the concomitant medication group demonstrated an IOP increase of 0.74 mmHg (Table 2).

The adverse events during the study were minimal. Conjunctival hyperaemia was presented in three patients, one from the fixed and two from the unfixed group. In addition, one patient from the unfixed group complained of itching and foreign body sensation.

## 4. Discussion

Glaucoma treatment is mainly based on achieving the individualised predetermined target IOP, with patient adherence to medication being a determinant factor [22,23]. Many studies tried to identify the causes of non-adherence to glaucoma treatment. The simplicity and easiness of therapy are likely to reduce the side effects and improve long-term tolerance to medication, with the fixed combination regimens showing a non-inferior effect on IOP reduction compared to multiple agents in individual bottles [19,24,25,26]. About 28% to 55% of glaucoma patients fail to adhere to their medication, resulting in poor IOP control and glaucoma progression [11,25].

Previous studies investigated the effect of switching to a combined medication without a washout period on patient compliance. Following a questionnaire survey, patients report that the Latanoprost/Timolol combination resulted in forgetting to administer eye drops less frequently [27]. The compliance to treatment in our study was evaluated with the MEMS Cap system, which has been previously used as the gold standard method to record reliability in clinical practice and research [28,29]. We assessed the level of compliance between group A, which received combined therapy once a day, and group B, which received single doses three times a day, with the compliance rate significantly higher in the first group. At the 3-month visit, the adherence rate in group A was 75.6%, considerably higher than 61.2% in group B. At the 6-month follow-up visit, the difference in the mean value of adherence between the two groups remained high, with 73% and 57.4% in groups A and B, respectively. The patients using eye drops three times a day showed poorer compliance than those once a day, contributing to inadequate IOP control and glaucoma under-treatment. However, Inoue et al. demonstrated that the IOP was maintained at the same level despite increased treatment compliance when prostaglandin analogues and beta-blockers shifted to the fixed combination of travoprost/Timolol [30]. Visual field loss and glaucoma under-treatment have also been associated with poor compliance and ineffective eye drop instillation [31].

The long-term use of glaucoma eye drops also plays a vital role in corneal surface health. About 60% of people on IOP-lowering treatment show signs and symptoms of OSD, whereas 66% of patients with severe OSD also have glaucoma. Patients with glaucoma or OHT under chronic topical treatment suffer from OSD more frequently than non-glaucoma and non-OHT individuals [19,32,33]. OSD is related to preservatives in glaucoma medication, such as benzalkonium chloride (BAK). Although BAK prolongs the lifespan of eye drops and reduces the infection risk [34], it has a toxic effect on the cornea and conjunctiva [34,35,36,37]. The cumulative effect of the long-term use of multiple eye drops in combination with the load of BAK increases the exposure to toxic agents, which is responsible for the OSD symptoms and a deterioration in the quality of life [38].

In the present study, the decrease in break-up time appears significantly higher in the unfixed group. The patients who received glaucoma eye drops once daily showed a BUT decrease of 6.5%, whereas those with eye drops three times daily demonstrated a a BUT reduction of 20.0%. Similarly, the corneal and conjunctival staining and the tear production level appeared to be improved in the patients with LTFC use once daily. Moreover, the break-up time is a parameter that has been proven to be significantly affected in newly diagnosed glaucoma patients treated with short-term fixed combination brinzolamide/Timolol. This indicates that tear film destabilisation is the first OSD parameter affected [39].

A study by Van Went et al. confirmed the hypothesis that the high prevalence of OSD is associated with the quality of a patient’s life and therapeutic management [40]. This has also been evaluated by a 6-month study, which assessed the impact of shifting from brinzolamide and Timolol concomitant medication to a fixed combination on the quality of life and ocular surface health. The fixed combination was proven effective and well-tolerated in terms of symptoms, whereas improving the quality of life could also explain the increased treatment compliance [41,42]. In addition, experimental rabbit models highlight that the fixed combination medication with a lower concentration of preservatives reduces the ocular surface changes [43].

The superiority of fixed combinations on the IOP control compared to monotherapy has been thoroughly investigated in the literature. A meta-analysis by Liu et al. demonstrated that the fixed combination of Latanoprost/Timolol is superior to Latanoprost monotherapy in lowering IOP [44]. Regarding the combination regimens, the Latanoprost/Timolol combination provides a significantly high IOP decrease among combination drugs containing Timolol [45,46]. Konstas et al. demonstrated that the fixed combination LTFC is a safe and effective regimen that reduces IOP equivalently when replacing the co-administered drug components [47]. However, the administration time seems to affect the hypotensive action of the fixed combination Latanoprost/Timolol regimen. When used in the morning, the mean IOP appears higher than the unfixed combination [48]. Our study proved that the fixed combination maintains the IOP in the first 3 months of treatment with a significantly increased hypotensive outcome at 6 months. The increased hypotensive result is related to improved patient adherence and the elimination of the flushing phenomenon. Inoue et al. also concluded that the hypotensive action was maintained, and compliance was enhanced at 1 and 3 months when dorzolamide and Timolol were shifted to the respective fixed combination without a washout period [30]. On the other hand, a double-blind, randomised multicentre study enrolled 190 patients with POAG and OHT, revealing that fixed combination LTFC, despite being safe, cannot adequately control the IOP [49].

Our study has some limitations to be considered when interpreting the results. The study evaluated the compliance of patients using the MEMS Cap system. However, patients were not trained on eye drop use, and we did not video-record the instillation technique and could not evaluate the consistency of eye drops use at home. In addition, the present study was conducted at a single centre, which carries some drawbacks to the specific characteristics of the population and the generalisability of the result to the broad population. One of the parameters that should be considered when interpreting the outcomes is the wide age range, although the median age is similar between groups. Some parameters related to the ocular surface appeared more often in older people, thus, the age variation within groups could affect our study results. Although the baseline characteristics of the study participants were similar and comparable between the two groups, it was not a randomised clinical trial. This carries a significant risk of bias with a limited ability to guarantee reliable results and the comparability of the intervention and control groups.

Despite these limitations, our study provides real-world results for the effectiveness of the single-dose treatment in ordinary circumstances. The fixed combination instead of two separate bottles provides fewer eye drop instillations and reduced the total dose over the day. The ease of the dosing regimen leads to better adherence with fewer ocular surface symptoms, improving the visual function and quality of life.

## 5. Conclusions

This study provides real-life evidence-based results for the superiority of fixed combinations in patient adherence and ocular surface health. The single eye drop use also showed a reasonable IOP control at all follow-up visits compared to the co-administration regimen. However, the results of our research will have to be confirmed with future studies and the outcomes implemented in daily clinical practice.

## Figures and Tables

**Table 1:** Demographics—baseline characteristics.

	Fixed Group	Unfixed Group
Number of Patients	71	71
Gender (%)		
Male	26 (36.6)	25 (35.2)
Female	45 (63.4)	46 (64.8)
Eye (%)		
RE	33 (46.5)	35 (49.3)
LE	38 (53.5)	36 (50.7)
Median Age (IQR)	68 (19)	65 (18)
Diagnosis (%)		
OHT	14 (19.7)	9 (12.7)
POAG	35 (49.3)	45 (63.4)
XFG	22 (31.0)	17 (23.9)
Pre-study Treatment Duration	4.3 (0.5)	4.5 (0.4)
BCVA	0.8 (0.3)	0.8 (0.4)
Median C/D ratio (IQR)	0.6 (0.25)	0.65 (0.25)
Median VF loss (IQR)	2.4 (4.8)	4.2 (6.8)
Mean CCT (SD)	532.56 (41.50)	537.73 (33.62)
Mean IOP (SD)	15.79 (2.81)	15.97 (3.44)
Median BUT (IQR)	6.0 (4.0)	4.0 (2.0)
Median Van Bijsterveld score (IQR)	1.0 (2.0)	2.0 (2.0)
Median Schirmer (IQR)	11.0 (9.0)	8.0 (5.0)

Abbreviations: RE, right eye; LE, left eye; IQR, interquartile range; OHT, ocular hypertension; POAG, primary open-angle glaucoma; XFG, exfoliation glaucoma; BCVA, best corrected visual acuity; C/D, Cup/Disc; VF, visual field; CCT, central corneal thickness; SD, standard deviation; IOP, intraocular pressure; BUT, break-up time.

**Table 2:** The study results.

Test	Fixed Group	Unfixed Group	*p*-Value
Adherence % (IQR)			
3 months	78.0 (33)	63.0 (37)	<0.001
6 months	73.0 (39)	58.5 (38)	0.003
Break-Up Time (IQR)			
Baseline	6.0 (4.0)	5.0 (2.0)	0.003
3 months	5.0 (3.0)	4.0 (2.0)	<0.001
6 months	5.0 (3.0)	4.0 (2.0)	<0.001
Schirmer Test I (IQR)			
Baseline	11.0 (9.0)	8.0 (4.0)	<0.001
3 months	11.0 (6.0)	8.0 (8.0)	<0.001
6 months	12.0 (6.0)	8.5 (8.0)	0.002
Van Bijsterveld score (IQR)			
Baseline	1.0 (2.0)	2.0 (2.0)	0.012
3 months	2.0 (2.0)	2.0 (3.0)	0.016
6 months	2.0 (2.0)	3.0 (2.0)	0.003
Intraocular Pressure (SD)			
Baseline	15.79 (2.81)	15.97 (3.44)	0.729
1 month	15.76 (3.12)	16.24 (3.50)	0.391
3 months	15.44 (2.40)	16.03 (2.90)	0.188
6 months	15.0 (2.41)	16.71 (4.56)	0.006

Abbreviations: IQR, interquartile range; SD, standard deviation.

## Data Availability

All data generated or analysed during this study are included in this article. Further enquiries can be directed to the corresponding author.
